# Reciprocity: Different behavioural strategies, cognitive mechanisms and psychological processes

**DOI:** 10.3758/s13420-019-00394-5

**Published:** 2019-11-01

**Authors:** Manon K. Schweinfurth, Josep Call

**Affiliations:** School of Psychology and Neuroscience, University of St Andrews, St Mary’s Quad, KY16 9JP St Andrews, Scotland

**Keywords:** Cooperation, Reciprocity, Cognition, Emotion, Norway rat, Vampire bat, Capuchin monkey

## Abstract

Reciprocity is probably one of the most debated theories in evolutionary research. After more than 40 years of research, some scientists conclude that reciprocity is an almost uniquely human trait mainly because it is cognitively demanding. Others, however, conclude that reciprocity is widespread and of great importance to many species. Yet, it is unclear how these species reciprocate, given its apparent cognitive complexity. Therefore, our aim was to unravel the psychological processes underlying reciprocity. By bringing together findings from studies investigating different aspects of reciprocity, we show that reciprocity is a rich concept with different behavioural strategies and cognitive mechanisms that require very different psychological processes. We reviewed evidence from three textbook examples, i.e. the Norway rat, common vampire bat and brown capuchin monkey, and show that the species use different strategies and mechanisms to reciprocate. We continue by examining the psychological processes of reciprocity. We show that the cognitive load varies between different forms of reciprocity. Several factors can lower the memory demands of reciprocity such as distinctiveness of encounters, memory of details and network size. Furthermore, there are different information operation systems in place, which also vary in their cognitive load due to assessing the number of encounters and the quality and quantity of help. We conclude that many species possess the psychological processes to show some form of reciprocity. Hence, reciprocity might be a widespread phenomenon that varies in terms of strategies and mechanisms.

## Introduction

The theory of natural selection predicts that only those behaviours evolve that increase the actor’s own survival and reproductive success (Darwin, 1859). Paradoxically, many species provide benefits to others, for instance, by providing care, food, information and support to con- and heterospecifics (Dugatkin, 1997). Cooperation is such a widespread phenomenon that we find evidence across the animal kingdom, ranging from bacteria (Crespi, 2001) to humans (Fehr & Fischbacher, 2003). The paradox of the evolution of cooperation was resolved for interactions between related individuals, i.e. by helping kin, shared genes are more likely to be transmitted to the next generations, which is in the interest of the helper (Hamilton, 1964). Still, the kin selection theory cannot explain the frequent occurrence of cooperation among unrelated individuals. Trivers (1971) offered a solution: reciprocal cooperation, i.e. helping those that were cooperative before. While several theoretical models have shown that cooperation can evolve via reciprocity (reviewed in Nowak, 2012), the theory has faced considerable resistance (e.g. Clutton-Brock, 2009; Connor, 2010; Hammerstein, 2003; Russell & Wright, 2009; Sánchez-Amaro & Amici, 2015; Stevens, Cushman, & Hauser, 2005; Stevens & Hauser, 2004, 2005; West, Griffin, & Gardner, 2007).

It is a central problem of the theory of reciprocity, and explaining cooperation more generally (especially among non-kin), that the extent to which behavioural exchanges are based on reciprocity is unknown. This problem is tightly linked to the cognitive underpinnings of reciprocity. Researchers, who assume reciprocity to be highly cognitively demanding, came to the conclusion that reciprocity is virtually absent in non-human animals (Amici et al., 2014; Clements & Stephens, 1995; Hauser, McAuliffe, & Blake, 2009; Pelé, Dufour, Thierry, & Call, 2009; Pelé, Thierry, Call, & Dufour, 2010; Sánchez-Amaro & Amici, 2015; Stephens, McLinn, & Stevens, 2002; Stevens et al., 2005; Stevens & Hauser, 2004). In contrast, researchers who assume that reciprocity varies in its cognitive load came to the opposite conclusion that reciprocity is widespread (Brosnan & de Waal, 2002; Brosnan, Salwiczek, & Bshary, 2010; Carter, 2014; Freidin, Carballo, & Bentosela, 2017; Melis & Semmann, 2010; Raihani & Bshary, 2011; Schino & Aureli, 2009, 2010b; Taborsky, Frommen, & Riehl, 2016). Here, we argue that the debate can be enriched and potentially resolved by identifying and discussing the concrete psychological processes of all different forms of reciprocity by using and incorporating findings from different lines of research. Although several authors have discussed the various mechanisms that may underlie reciprocal exchanges between individuals (e.g. Brosnan & de Waal, 2002; Schino & Aureli, 2010b), as far as we know, no attempt has been made to systematically relate those mechanisms to the psychological processes underlying them. Our aim in this article is to bring attention to this issue as a necessary step towards the ultimate goal of elucidating the psychological processes underlying different forms of reciprocity. Accordingly, our article is organised as follows: First, we summarise different behavioural strategies and cognitive mechanisms enabling reciprocity. Second, we apply this framework to three textbook examples of reciprocity, i.e. the Norway rat (*Rattus norvegicus*), the common vampire bat (*Desmodus rotundus*) and the brown capuchin monkey (*Cebus apella*). Third, we discuss the crucial behavioural, cognitive and emotional components enabling different forms of reciprocity. These psychological processes will allow us to draw clear predictions under which conditions reciprocity is likely to evolve. Finally, we provide concrete examples for prospective studies to better understand the evolutionary and psychological origins of reciprocity.

## Different forms of reciprocity

Trivers (1971) defined reciprocity as one individual selectively providing helpful acts with another individual that will provide benefits in return. Hereby, helpful acts are costly to the actor and beneficial only to the recipient. There are various examples of evidence for individuals that exchange help conditional on the partner’s previous helping level across the animal kingdom (reviewed in Díaz-Muñoz, DuVal, Krakauer, & Lacey, 2014; Dugatkin, 1997; Hemelrijk & Luteijn, 1998; Jaeggi & Gurven, 2013; Schino, 2007; Schino & Aureli, 2008, 2010c; Taborsky, 1994; Taborsky et al., 2016). Evidence ranges from bacteria (Inglis, West, & Buckling, 2014) to humans (Bowles & Gintis, 2011). However, such conditional help has not always been termed *reciprocity* because some authors suggest restricting the term to situations in which individuals are able to cognitively compute cost-benefit analyses (reviewed in Carter, 2014).

We think restricting the term *reciprocity* to complex computations, i.e. keeping track and weighing costs and benefits when helping other, is problematic for at least three reasons. First, humans, who are often regarded as the only species capable of reciprocity, rarely reciprocate favours by calculating costs and benefits (Bowles & Gintis, 2011, pp. 186-194; Carter, 2014). Furthermore, when defining reciprocity, Trivers did not restrict the term to animals that are capable of calculating such cost-benefit analyses. In fact, he suggested that emotions might be guiding human reciprocity (Trivers, 1971). He proposed that (1) sympathy might be the motivator to help, (2) gratitude the measure of helpfulness, (3) guilt the reason to not cheat and (4) trust the mechanism of partner choice. Restricting the term reciprocity to complex calculations would hence also restrict human reciprocity to only a few situations, i.e. mainly business relationships, which does not resemble the everyday usage of the term (cf. Cambridge Dictionary). Second, restricting the term to complex calculations prevents us from understanding the general explanatory power of the theory of reciprocity. Many animals cooperate conditionally with kin and non-kin (see above). However, these results have been largely neglected because the focus of the current debate lies on explaining why reciprocity is rare or cannot exist (e.g. André & Nolfi, 2016; Hammerstein, 2003; Russell & Wright, 2009). Third, restricting reciprocity to few instances prevents us also from understanding its evolutionary origins. Studying conditional cooperation in various species may eventually inform us about important questions, such as how many times reciprocity evolved, under which condition it evolved and how it is maintained.

For these reasons, we define reciprocal interactions as “individuals exchanging goods and services that are contingent on each other”, according to Trivers’ original definition (Trivers, 1971). This definition makes no assumptions about the underlying mechanisms. Instead, this definition allows us to investigate different mechanisms enabling individuals to reciprocate (please see our glossary in the Online Supplementary Material, S1, for different forms of reciprocity). Importantly, different lines of research have focussed on different aspects of reciprocity, which have not yet been aligned, although they can greatly inform each other, as we outline below.

One line of research has focussed mostly on describing conditional strategies in the framework of reciprocity (e.g. Axelrod & Hamilton, 1981; Taborsky et al., 2016; West, El Mouden, & Gardner, 2011). A strategy is a behavioural decision rule that is dependent, in the case of reciprocity, on the partner’s behaviour (cf. Bshary & Bergmüller, 2008; Laland, 2004). There are at least three forms of reciprocal strategies if by-products are excluded, like pseudo-reciprocity, i.e. an automated response to help without the possibility of cheating (Connor, 1986). First, in *direct reciprocity*, individuals help only those that helped them previously, i.e. “I help you because you helped me” (Trivers, 1971). Second, *indirect reciprocity* relies on public information where individuals help individuals that were observed to be helpful towards others, i.e. “I help you because you helped someone else” (Alexander, 1987). Third, *generalised reciprocity* is based on a general increase in the motivation to help due to a random experience of help, i.e. “I help you because I was helped by someone” (Boyd & Richerson, 1989). While these behavioural strategies describe useful means that can be tested empirically and theoretically, they give us little insight into how such rules are achieved.

Another line of research has focussed mostly on mechanisms that may enable reciprocity (e.g. Brosnan & de Waal, 2002; Schino & Aureli, 2010b). A mechanism describes the basal cause of a behaviour or a strategy, which can be cognitive in the case of reciprocity (Bateson & Laland, 2013; Tinbergen, 1963). At least four mechanisms have been described, if again by-products are excluded, like symmetry-based reciprocity, i.e. a response based on symmetrical traits rather than experienced help (de Waal & Luttrell, 1988; but see Campennì & Schino, 2016). First, probably the least cognitively demanding form of reciprocity is *hard-wired reciprocity* (Schino & Aureli, 2017). This is a fixed response to provide help immediately upon the receipt of help. It is most likely confined to distinct settings that do not involve a time delay, partner choice or the flexible use of different commodities. Thus, this mechanism is different from all other mechanisms that are flexible and require some form of encoding and processing information. Second, *attitudinal reciprocity* requires a cognitive decision with a minimum of information. Here an attitude, i.e. a tag based on the last encounter, is associated with a cooperation partner (Brosnan & de Waal, 2002; de Waal & Brosnan, 2006). This mechanism does not require the memory of the exact levels of help. Instead a general tag, such as “the partner was nice” is sufficient. An important feature of attitudinal reciprocity is that received help will be reciprocated in the next cooperative situation, i.e. short-term reciprocity (Jaeggi, de Groot, Stevens, & van Schaik, 2013). Third, *emotion-based reciprocity* acts on longer time spans. For that, individuals help selectively those partners with which they associate positive emotions, for example when being socially bonded (Schino & Aureli, 2009). Because emotional bonds develop as a consequence of help received, the donated help is likely to be returned. This mechanism leads to balanced relationships in which given and received favours are correlated over long time frames, i.e. long-term reciprocity. Finally, *calculated reciprocity* is probably the most cognitively demanding mechanism because individuals calculate how much they need to help another individual based on the memory of the amount of help received by this individual (Brosnan & de Waal, 2002; de Waal & Brosnan, 2006). Similar to attitudinal reciprocity, calculated reciprocity leads to short-term reciprocity in which provided help is dependent on previously received help.

These different forms of reciprocity illustrate that there is probably not ‘one form’ of reciprocity (Table 1; see also the glossary Online Supplementary Material, S1). Yet, many studies did not differentiate between them; consequently, we thus lack knowledge on the use of different strategies and mechanisms in many species.

**Table 1:** Overview of different reciprocity types

	Catch phrase	Strategy (time scale)	Information	Processing	Species example
Hard-wired reciprocity	I help you because donations immediately and automatically follow gifts	Generalised, direct reciprocity (immediate)	/	No processing involved	Plants and their mycorrhizal fungal symbionts
Attitudinal reciprocity	I help you because someone did something nice to me	Generalised reciprocity (short-term)	What?	Substitution	Norway rats, brown capuchin monkeys
	I help you because you did something nice to me/others	In-, direct reciprocity (short-term)	Who? / What? / To whom?	Substitution	Norway rats, brown capuchin monkeys
Emotion-based reciprocity	I help you, because I like you	Direct reciprocity (long-term)	Who? / Associated emotion?	Accumulating	Common vampire bats, brown capuchin monkeys
Calculated reciprocity	I help you because you helped me XY times over XY encounters, worth the value XY	(In-), direct reciprocity (short-term)	Who? / What? / When? / How much? / To whom? / (…)	Computing	Adult humans

## Three textbook examples of reciprocity

Next, we examine evidence of different forms of reciprocity in the light of their behavioural strategies and cognitive mechanisms in three textbook examples, i.e. Norway rats, common vampire bats and brown capuchin monkeys (summarised in the Online Supplemental Material, Table S2). We chose these examples as they provide the most comprehensive data sets of reciprocity in comparison to other species. At the same time, these studies have also attracted criticism and doubts have been raised whether the species’ ability to reciprocate might be accounted by alternative explanations (e.g. Davies, Krebs, & West, 2012; McAuliffe & Thornton, 2015; Paolucci, Conte, & Di Tosto, 2006; Stevens & Hauser, 2005; Zentall, 2015). In recent years, however, initial findings in these species have been validated, refined and extended by using various paradigms and by testing the raised concerns (see below). Thereby, these three species have become the best investigated study systems of reciprocity to date. Therefore, we think they provide a useful starting point of investigating the evolutionary and psychological origins of reciprocity.

### Norway rat

Wild Norway rats live in large multi-female, multi-male colonies, which may be composed of more than 150 individuals (Davis, 1953). They frequently interact with related and unrelated colony members and form dominance hierarchies (Calhoun, 1979). They engage in various social behaviours like alarm calls, food sharing, huddling, social grooming and social play (reviewed in Schweinfurth, under review).

Norway rats have been repeatedly shown to reciprocate help in different paradigms (reviewed in Schweinfurth in press). The most commonly used paradigm is the food-exchange setup. Here one rat, i.e. the cooperating partner, provides food via a movable platform to the focal rat (cf. Rutte & Taborsky, 2007). After a delay of up to six days, the roles are exchanged and the focal rat can provide food to its previous partner (e.g. Stieger, Schweinfurth, & Taborsky, 2017). To ensure that food donations by the focal rats are based on the previous help by a partner, focal rats are always also tested with a defecting partner that did not provide food to them. Several controls have been conducted to ensure that, for instance, differential food intake, activity, copying, or other factors cannot explain their helping levels (for a discussion, see Dolivo, Rutte, & Taborsky, 2016; Schweinfurth & Taborsky, 2018b).

Rats help each other reciprocally according to at least two behavioural strategies. First, they help each other according to direct reciprocity. Female and male rats donate food to others reciprocally by providing more food to cooperating than defecting partners (Li & Wood, 2017; Rutte & Taborsky, 2008; Schneeberger, Dietz, & Taborsky, 2012; Schweinfurth, Aeschbacher, Santi, & Taborsky, 2019; Schweinfurth & Taborsky, 2016, 2017, 2018c; Simones, 2007; Viana, Gordo, Sucena, & Moita, 2010). In addition, female rats apply direct reciprocity when grooming each other (Schweinfurth, Stieger, & Taborsky, 2017). Such reciprocal allogrooming has significant fitness benefits, as reciprocal groomers live longer and suffer less mammary tumours in the lab (Yee, Cavigelli, Delgado, & McClintock, 2008). Finally, female rats also exchange allogrooming for food and vice versa (Schweinfurth & Taborsky, 2018b; Stieger et al., 2017). Besides direct reciprocity, female, but not male, rats engage in generalised reciprocity (Rutte & Taborsky, 2007, 2008; Schweinfurth et al., 2019). In a direct comparison, however, female rats donate over 20% more food to a partner with whom they have interacted, showing that direct reciprocity generates higher levels of cooperation than generalised reciprocity (Rutte & Taborsky, 2008).

What is the mechanism underlying reciprocity in Norway rats? First, hard-wired reciprocity cannot explain reciprocity among Norway rats because they tailor their help to the partner’s helping quality (Dolivo & Taborsky, 2015) and the partner’s need (Márquez, Rennie, Costa, & Moita, 2015; Schneeberger et al., 2012; Schneeberger, Röder, & Taborsky, submitted; Schweinfurth & Taborsky, 2018a). Furthermore, rats reciprocate help by using different actions (Schweinfurth & Taborsky, 2017) and different commodities (Schweinfurth & Taborsky, 2018b; Stieger et al., 2017), making a fixed response unlikely. Second, emotion-based reciprocity is also unlikely to explain reciprocity in Norway rats. They seem not to form social bonds even after being housed together for more than one and a half years (Schweinfurth, Neuenschwander, et al., 2017). They do not accumulate social information with their partners, but rather use the last experience (Schweinfurth & Taborsky, under review; Stieger et al., 2017). Third, there is no evidence for calculated reciprocity either. The amounts of received and immediate given help are not matched in male and female rats (Schweinfurth et al., 2019; Schweinfurth & Taborsky, under review). This is in line with their numerical ability being limited to six items, which is below the amount of grooming bouts they are known to reciprocate (cf. Davis & Bradford, 1986; Schweinfurth, Stieger, et al., 2017).

Reciprocity in Norway rats is probably best explained by attitudinal reciprocity (reviewed in Schweinfurth, in press). Rats form attitudes that are based on the last encounter with a partner (Schweinfurth & Taborsky, under review). Importantly, using the last encounters for reciprocity is not a result of memory interference as rats have been shown to memorise several partners (Kettler, Schweinfurth, & Taborsky, under review), several food preferences (Galef, Lee, & Whiskin, 2005) and several unique events (Panoz-Brown et al., 2016) despite a long time delay with potentially disruptive experiences. Importantly, attitudes are linked to received cooperation and not just the product of ‘feeling good’, as a study showed in which rats refused to reciprocate food donations that they received in the presence of, but not by, another rat (Schmid, Schneeberger, & Taborsky, 2017). Attitudes can be generalised to other partners because female rats show not only direct but also generalised reciprocity (Rutte & Taborsky, 2008). In addition, attitudes are not binary responses, like a cooperator or defector tag, but can be modulated by different values of help. For instance, rats groom a partner more often that has provided food to them than vice versa, suggesting that one food item is valued more than one allogrooming bout (Schweinfurth & Taborsky, 2018b). In addition, rats are more likely to provide oat flakes to a partner that provided them with banana pieces, i.e. highly preferred food, than with carrot pieces, i.e. less preferred food (Dolivo & Taborsky, 2015).

### Common vampire bat

Vampire bats, which include three species, feed exclusively on the blood of other mammals (Dalquest, 1955). Common vampire bats live in small groups of eight to twelve individuals (Wilkinson, 1984). Such groups roost together in large colonies, ranging from a few individuals to over 2,000, whereby females often move between different roosts (Wilkinson, 1988). Groups usually consist of one male and its female harem or male bachelor groups (Wilkinson, 1985b, 1985a). Vampire bats are the only bats that regurgitate food to donate it to others (Carter & Wilkinson, 2013a). Besides food donations, vampire bats show high levels of allogrooming compared to other bats (Carter & Leffer, 2015). In addition, they adopt and nurse offspring of other colony members (Carter & Wilkinson, 2013a; Wilkinson, Carter, Bohn, & Adams, 2016).

Common vampire bats regurgitate blood to donate it to others. Early studies used numerous observations of these bats in their natural habitat and found that given and received help is correlated and reciprocated (Denault & McFarlane, 1995; Wilkinson, 1984). Recently, these findings have been replicated (Carter & Wilkinson, 2013c) and extended by several controlled experiments with captive bats (reviewed in Carter & Wilkinson, 2013b). In several experiments, individual focal bats were removed from their colony and fasted for one day. After the hungry focal individual was returned to its group, all food donations to this individual were recorded. The focal individuals received blood mostly from partners, which they had provided food before (Carter & Wilkinson, 2013c).

They mainly donate blood with conspecifics with which they roost together frequently but that do not necessarily belong to their group (Carter & Wilkinson, 2013b). Food donations are more common between females, which form stable social bonds, but males have also been shown to regurgitate blood for others in the laboratory (Carter & Wilkinson, 2013b). Such donations are highly valuable to recipients because vampire bats die within two to three days without a blood meal (Freitas, Welker, Millan, & Pinheiro, 2003; McNab, 1973). They reciprocate blood with both kin and non-kin, whereby donations are better explained by reciprocity than by relatedness (Carter & Wilkinson, 2013c). Extending the network to non-kin has been shown to be beneficial for the bats because when their main association partner was temporarily removed, those that had more associations with unrelated roost mates received more food donations (Carter, Farine, & Wilkinson, 2017; Carter & Wilkinson, 2015b). Besides exchanging blood, the bats also exchange allogrooming for food according to direct reciprocity (Carter & Wilkinson, 2013c; Wilkinson, 1986). In contrast, they seem not to use generalised reciprocity (Carter & Wilkinson, 2013c).

What is the mechanism underlying reciprocity in common vampire bats? First, hard-wired reciprocity cannot explain reciprocity since the bats exchange different services, like allogrooming with food (Carter & Wilkinson, 2013c). In addition, reciprocity is limited to few closely bonded individuals (Carter et al., 2017), which makes automatic responses to received help unlikely. Second, attitudes based on recent encounters seem to be unable to generate reciprocity because reciprocity can only be detected over long time frames or when socially bonded bats are observed in captivity (reviewed in Carter & Wilkinson, 2013b; Wilkinson et al., 2016). Third, calculated reciprocity is an unlikely explanation for their reciprocity. Like Norway rats, common vampire bats do not match the actual amount of received and given help (Carter & Wilkinson, 2013c), which makes reciprocity based on calculations unlikely. However, little is known about their cognitive capabilities that could elucidate the underlying cognitive processes that they use (for the only studies of examining cognitive skills, see Ratcliffe, Fenton, & Galef, 2003; Vrtilek, Carter, Patriquin, Page, & Ratcliffe, 2018).

Reciprocity in vampire bats is probably best explained by emotion-based reciprocity. Rather than considering the last encounters as Norway rats do, the bats reciprocate help over long time spans (see above). They form enduring social bonds with kin and non-kin, which can last over more than ten years (Carter et al., 2017; Carter & Wilkinson, 2013c, 2015b; Wilkinson, 1985b, 1985a, 1986). Importantly, their food-sharing network closely mirrors their social-bonding network (Carter & Wilkinson, 2013c). This suggests that social bonds play a crucial role in their decisions to help conspecifics. In line with this, the amounts of donated blood and grooming are linked to oxytocin levels, which suggests an emotional component in the decision to reciprocate (Carter & Wilkinson, 2015a).

### Brown capuchin monkey

Brown capuchin monkeys, also called tufted or black-capped capuchin monkeys, live in colonies of up to 30 individuals (Carosi, Linn, & Visalberghi, 2005). They show a distinct linear hierarchy in both sexes (Janson, 1985). Further, their breeding system can be described as either one-male, multi-female or multi-male, multi-female (Carosi et al., 2005). These monkeys show various social behaviours, such as alarm calls, collaborative hunting, food sharing, social grooming and social play (Fragaszy, Visalberghi, & Fegan, 2009; Izawa, 1980).

Brown capuchin monkeys are probably the best-known example for reciprocity. Reciprocity has been demonstrated repeatedly by using various methods under controlled captive conditions. For instance, they donate food to others by handing over or dropping food close to a partner in an adjacent compartment, who will return the favour in the same way (de Waal, 2000; see de Waal, 1997, for a detailed ethogram of the donations). To ensure that food donations are a product of received help and partner directed, several control conditions have been conducted with partners of differing relationship quality or partners being absent (reviewed in de Waal & Brosnan, 2006). In addition to these active and passive food transfers, the monkeys also reciprocate food donations by using various less intuitive testing apparatuses, i.e. bar pulling, joysticks, lever boxes and token exchanges (Hattori, Kuroshima, & Fujita, 2005; Mendres & de Waal, 2000; Parrish, Brosnan, & Beran, 2015; Suchak & de Waal, 2012).

All the tests described above investigated direct reciprocity. In addition, capuchin monkeys show generalised reciprocity (Leimgruber et al., 2014). As far as we are aware, there has been no published report of indirect reciprocity in brown capuchin monkeys. However, they pay attention to third-party interactions and are inclined to accept exchange offers from humans that were observed to frequently reject such exchange requests from other monkeys (Anderson, Kuroshima, Takimoto, & Fujita, 2013). This might suggest indirect reciprocity, but further studies are needed that directly test this possibility.

What is the mechanism underlying reciprocity in brown capuchin monkeys? In contrast to Norway rats and common vampire bats, the mechanism of reciprocity among these monkeys is less obvious. The only mechanism that can be almost certainly excluded is hard-wired reciprocity. When the monkeys help each other, they consider the quality of received help (de Waal, 2000) and the quality of their relationship (Sabbatini, De Bortoli Vizioli, Visalberghi, & Schino, 2012). This makes a fixed response unlikely. Furthermore, calculated reciprocity seems unlikely but possible because two studies found that the amount of received and given help was correlated, which suggests memory and score keeping of previous helpful events to some degree (e.g. de Waal, 1997, 2000). In fact, capuchin monkeys have been shown to add items to a pool of non-visible items, suggesting that they can keep track of multiple food donations, which is needed for calculations (Beran, Evans, Leidghty, Harris, & Rice, 2008). However, not all studies found such a correlation between received and given help (Brosnan, Freeman, & de Waal, 2006; Sabbatini et al., 2012; Suchak & de Waal, 2012). Finally, studies set out to test for calculated reciprocity found no evidence (Amici et al., 2014; Pelé et al., 2010). It should be noted, however, that the monkeys in these studies did not pass the task-understanding condition. Therefore, additional studies on calculated reciprocity are needed.

There is less ambiguous evidence for the two remaining mechanisms underlying reciprocity. There is good evidence for attitudinal reciprocity (reviewed in Brosnan & de Waal, 2002; de Waal & Brosnan, 2006). In contrast to common vampire bats and in accordance with Norway rats, brown capuchin monkeys show short-term reciprocity (see above). Furthermore, they reciprocate help with familiar and unfamiliar partners (Suchak & de Waal, 2012), which suggests short-term attitudes rather than long-term bonds being the reason to help. However, there is also good evidence for emotion-based reciprocity. In a direct comparison, the monkeys were more likely to allow access to their food to a socially bonded individual than to an individual that provided food to them before (Sabbatini et al., 2012). This study suggests that the monkeys probably cooperate over long time frames and an emotional bond can become more important that attitudinal reciprocity in a partner choice situation. This is in line with observations from the wild that showed evidence for long-term reciprocity (di Bitetti, 1997; Izawa, 1980; Schino, di Giuseppe, & Visalberghi, 2009a; Schino, Di Giuseppe, & Visalberghi, 2009b; Tiddi, Aureli, Polizzi di Sorrentino, Janson, & Schino, 2011; Tiddi, Aureli, & Schino, 2012).

Hence, reciprocity in brown capuchin monkeys is probably a result of attitudinal reciprocity and emotion-based reciprocity. While attitudinal reciprocity cannot explain the selective helping of bonded individuals, emotion-based reciprocity cannot explain the repeatedly observed short-term reciprocity. Still, the results must not be contradictory, and both may explain some aspects of cooperation in these monkeys. For instance, Sabbatini et al. (2012) found that the same monkeys show short-term reciprocity in dyads, but long-term reciprocity in trios. This is an interesting finding because it suggests that individuals can possess multiple mechanisms to achieve reciprocity. The same may apply to other species, although this has not been studied extensively other than in humans.

Humans use different mechanisms to achieve different forms of reciprocity. People employ a calculated reciprocity approach with unfamiliar (Andreoni & Miller, 1993; Gachter & Falk, 2002) or business partners (Anderson, Hakansson, & Johanson, 1994; Steidlmeier, 1999). If, however, it gets difficult to memorise several events with a partner, people focus on the attitude towards partners based on the last encounter (Milinski & Wedekind, 1998). In contrast, we rarely use short-term reciprocity when interacting with friends; instead, we rely on emotional bonds based on long-term reciprocity (reviewed in Massen, Sterck, & de Vos, 2010; Silk, 2003). Overall, we help friends more than strangers (Gächter, Kessler, & Königstein, 2011) because we trust them (Buchan, Croson, & Dawes, 2002; Majolo et al., 2013) and expect them to return favours (Deutsch, 1975; Walker, 1995). Interestingly, donations towards strangers can be increased by applying oxytocin, which is associated with emotional bonds (Zak, Stanton, & Ahmadi, 2007). In addition, the more often strangers interact, the more trust is built up and the more they are treated like friends (Gächter et al., 2011). This suggests that we use multiple mechanisms that can transform into each other rather than being static.

## Psychological processes of reciprocity

We have shown above that different species show different strategies that are enabled by different mechanisms. The psychological processes of calculated reciprocity have been discussed at length (e.g. Hauser, McAuliffe, & Blake, 2009; Ramseyer, Pelé, Dufour, Chauvin, & Thierry, 2006; Russell & Wright, 2009; Sánchez-Amaro & Amici, 2015; Stevens, Cushman, & Hauser, 2005; Stevens & Gilby, 2004; Stevens & Hauser, 2004, 2005). Stevens and colleagues (2004, 2005) identified numerous possible cognitive challenges involved in reciprocity in order to direct the right amount of help to the correct partner. These challenges may include cheater detection, cheater punishment, cost-benefit computations, inhibitory control, individual recognition, memory capacity, memory decay, memory interference, numerical quantification, reputation recollection, temporal discounting, theory of mind and time estimation. In contrast, the psychological processes of other mechanisms have received uneven attention, even though several authors have pointed out that reciprocity is not necessarily cognitively demanding (e.g. Brosnan & de Waal, 2002; Schino & Aureli, 2009). In fact the overemphasis on calculated reciprocity is probably the reason why it is commonly assumed that reciprocity requires high cognitive demands and thus is unlikely to occur in non-human animals (see also Schino & Aureli, 2010a). Although this is the case of calculated reciprocity, it does not apply to reciprocity in general. Furthermore, different mechanisms have been largely neglected in studies investigating different strategies and vice versa, which is unfortunate because they can greatly complement and inform each other. Hence in this part, we aim to integrate the different approaches in one framework and highlight the psychological processes underlying different reciprocity forms (summarised in Table 2). To understand the psychological processes of reciprocal decisions, it is important to note that the most basal components of such decisions are memory, i.e. the encoding and retrieving of information, and operations, i.e. processing the information to provide help.

**Table 2:** Psychological processes associated with different reciprocity types

	Strategy	Perception	Scene-memory	Individual recognition	Emotions	Calculations	Event-memory
Hard-wired reciprocity	Direct, generalised	✓					
Attitudinal reciprocity	Generalised	✓	✓				
	Direct, indirect	✓	✓	✓			
Emotion-based reciprocity	Direct	✓		✓	✓		
Calculated reciprocity	Direct, (indirect)	✓	✓	✓	(✓)*	✓	✓

*Emotions are not a pre-requisite for calculated reciprocity but may be an enhancing factor

### Memory

If receiving and giving help takes place at the same time, e.g. simultaneous exchanges (MacDonald, Stewart, Stopka, & Yamaguchi, 2000), or is a result of each other, e.g. immediate exchanges (Kiers et al., 2011), no information needs to be stored and memory is not essential. This is, for instance, the case for hard-wired reciprocity and shows that memory is not a prerequisite for reciprocity *per se*. In contrast, if there is a delay between received and given help, memory is involved. All reciprocity forms besides hard-wired reciprocity, are likely to involve a time delay and thus require some form of memory. The longer the delay, the longer information needs to be stored and the more likely such information is modified or forgotten (Lind, Enquist, & Ghirlanda, 2015).

Memory has been suggested to constrain reciprocity in many species (reviewed in Stevens et al., 2005; Stevens & Hauser, 2004). For instance, the capacity to memorise several partners and their cooperativeness over long intervals can be a cognitive burden (Stevens, Volstorf, Schooler, & Rieskamp, 2011). Indeed, in a calculated reciprocity system, many details of several encounters need to be associated to specific individuals in order to make an informed decision to help. Such information is likely subject to memory decay due to delay or distractions (e.g. Milinski & Wedekind, 1998). In addition, interference, i.e. new information interferes with older information, must be reduced.

In contrast to calculated reciprocity, attitudinal reciprocity is tightly linked to this interference effect. Here, information is always replaced by newer information, or in other words, the most recent encounter is memorised better than previous encounters, i.e. recency effect (Raffel, 1936). Attitudes can be acquired by classical conditioning. If an individual helps a partner that reciprocates help, this partner might be encoded as a conditioned stimulus because it provides rewarding help. It is currently unclear how many partners in association with the respective attitude can be remembered. For example, pigeons (*Columba livia*) have been shown to memorise 800–1,200 associations, while baboons (*Papio papio*) could memorise at least 3,500–5,000 associations after intensive and extended training (Fagot & Cook, 2006). These results suggest that attitudinal reciprocity, which relies on simple associations between a partner and an attitude, might lie within the cognitive skillset of many animals even if several partners need to be encoded. Potentially, both calculated and attitudinal reciprocity might require even fewer memory demands when interactions require only the memory of the level of cooperation (generalised reciprocity), instead of the partner’s identity associated with their level of cooperation (direct and indirect reciprocity).

Furthermore, in emotion-based reciprocity, memory demands are even less likely to constrain reciprocity. First, the amount of information that needs to be stored about specific cooperation events can be drastically reduced, if individuals base their decisions on emotions associated with a partner because no information about specific encounters is needed (Schino & Aureli, 2017). In fact, the emotional bond with a partner might become an incentive cue. Bonds are based on repeated cooperation and thus individuals might associate received help with the bond strength. Eventually, helping closely bonded partners might be perceived as more rewarding than helping loosely bonded partners, which is a similar effect to food tasting better when hungry, i.e. incentive learning (reviewed in Berridge, 2001). Second, emotions have been shown to enhance memory (Dolan, 2002; Kensinger, 2004). For example, chimpanzees (*Pan troglodytes*) are better at remembering pictures that show emotional scenes, such as fighting, compared to neutral scenes, such as resting (Kano, Tanaka, & Tomonaga, 2008). In addition, American crows (*Corvus brachyrhynchos*) can remember dangerous people after a single fearful encounter for more than two and a half years (Marzluff, Walls, Cornell, Withey, & Craig, 2010). Finally, common ravens (*Corvus corax*) distinguish non-affiliated from affiliated conspecifics, with which they have had a positive emotional bond, even after 3 years of separation (Boeckle & Bugnyar, 2012). There have been some pioneering studies investigating the interplay of cooperation and emotions. For instance, chimpanzees that receive allogrooming or food show an increase in urinary oxytocin (Crockford et al., 2013; Wittig et al., 2014). Oxytocin is a hormone that has been frequently linked to positive emotions and increases generosity and reciprocity also in humans (Kosfeld, Heinrichs, Zak, Fischbacher, & Fehr, 2005; Zak et al., 2007). Recent findings suggest, however, that the increase in cooperativeness due to oxytocin may depend on the individual’s baseline levels and social contexts (Bartz, Zaki, Bolger, & Ochsner, 2011; Crockford, Deschner, Ziegler, & Wittig, 2014; Romero, Onishi, & Hasegawa, 2016). Still, if help is associated with emotions, the memory of such encounters might be enhanced. This may explain why many species can reciprocate help over extended periods of time without memory constraints (Freidin et al., 2017; Jaeggi et al., 2013). It may also explain why common ravens, for instance, are capable of remembering short prosocial encounters with humans for more than a month (Müller, Massen, Bugnyar, & Osvath, 2017). In addition to emotions, other factors are known to increase or decrease memory, which we discuss below.

### Memory and distinctiveness

Helpful acts may be easier to memorise when they involve emotional (see above), dramatic, rare or surprising situations rather than routine situations. For example, we are better able to recall the dinner menu we had last year in an exquisite restaurant compared to what we cooked one week ago. Likewise, individuals may better remember that they received support in a rare fight than hearing yet another alarm call.

To the best of our knowledge, distinctiveness has not yet been investigated in helping tasks, albeit evidence has been demonstrated in other tasks. Pigeons, for instance, are less likely to learn stimuli that are similar to each other and forget them more quickly compared to distinctively different stimuli (Kraemer, 1984). Similarly, apes and Norway rats memorise more easily distinct compared to indistinct stimuli (e.g. Beran, 2011; Lewis, Call, & Berntsen, 2017; Reed & Richards, 1996).

Distinctiveness of encounters is likely to be of great importance for reciprocity forms that depend on exact information of last encounters, such as calculated and attitudinal reciprocity. In contrast emotion-based reciprocity is not dependent on single encounters and therefore, distinctiveness is less likely to be a pre-requisite of this form. Rather, several cooperative events are accumulated into an emotional score on which the decision to help is based. Such encounters may involve customary activities, such as allogrooming, that strengthen the social bond (cf. Henzi & Barrett, 1999). Likewise, distinctiveness is probably of greater importance to direct and indirect reciprocity as more information needs to be memorised in comparison to generalised reciprocity.

### Memory and level of details

The level of details required for helping decisions may influence reciprocity. The more details are needed to make an informed decision to help others (e.g. who, what, when, to whom, when and how much/how often), the more information needs to be stored, which is likely to be constrained by memory capacity decay and interference. Furthermore, the more details need to be encoded, the more likely episodic memory is involved (Baddeley, 2001; Crystal, 2010; Tulving, 1972). This is a special form of memory that has been extensively studied in jays and rats and suggested only for few other species (Dere, Kart-Teke, Huston, & De Souza Silva, 2006). Rats might use episodic memory when reciprocating help (cf. Crystal, 2018). Rats can remember several partners over at least 4 days and remember the specific cooperating experience in combination with the partner’s identity (Kettler, Schweinfurth, & Taborsky, under review). However, future studies are needed to investigate the role of episodic memory in reciprocity among Norway rats.

As discussed in the previous sections, only calculated reciprocity relies on complex details of cooperative events and thus seems to require episodic memory. In attitudinal and emotion-based reciprocity, it seems more likely that help is encoded in scenes. Memory of such scenes requires a minimum of details of one or several events and no mental time travel (Rubin & Umanath, 2015). This form of memory has been shown to be involved in decisions of apes and zebra finches (*Taeniopygia guttata)* (Larose & Dubois, 2011; Lewis et al., 2017). The details of scenes can be further reduced to a simple association between an individual and a rewarding act, cooperation, which is needed for direct and indirect reciprocity. Here, only the valence of an encounter or a partner, i.e. more or less positive, is encoded. Almost all species studied have been shown to learn such associations (Olmstead & Kuhlmeier, 2015a) and thus may qualify for some forms of reciprocity. Probably the most extreme example that illustrates how few memorised details can lead to reciprocity is when attitudinal reciprocity underlies generalised reciprocity. In this situation, the partner’s identity is unimportant and decisions to help can be solely based on the valence of the most recent encounter.

### Memory and network size

Reciprocity is dependent on the network size (Hizak, Dmitrovic, & Cubrilo, 2018). While it seems easy to remember what one partner did over a given period, keeping track of several partners seems more challenging. Indeed in lab experiments, people show an increasing error rate, the more partners need to be remembered, leading to an error rate of over 20% when trying to keep track of only ten partners over several rounds (Stevens et al., 2011). This is an important finding, given that theoretical models demonstrated that reciprocity is unlikely to evolve, if individuals show error rates of only 2% in small groups of four individuals (Bowles & Gintis, 2011, pp. 65-66).

At the same time, these findings suggest that either memory for several partners can be increased or that reciprocity can be explained by mechanisms other than calculated reciprocity. Otherwise, we could not explain why reciprocity evolved in humans because early hominid hunter-gatherer groups already encompassed more than 30 people (Marlowe, 2005). In addition, contemporary humans have a social network size of around 150 individuals (Hill & Dunbar, 2003), which would make reciprocity basically impossible according to the above described estimations. There are at least three possibilities that may enable reciprocity in large groups.

First, memory demands can be lowered to allow cooperation with several partners (reviewed in Moreira et al., 2013). For example, humans in such lab-based games develop alternative strategies, where they only remember rare or distinct partners, for example remembering the only cheater in a pool of cooperators (Volstorf, Rieskamp, & Stevens, 2011). Second, instead of memorising exact helping levels, individuals may choose to cooperate with partners that helped them and refuse to interact with partners that did not help them, i.e. partner choice. Importantly, the bigger groups are, the more likely such partner choice evolves (Kurvers, Krause, Croft, Wilson, & Wolf, 2014). This would eventually lead to emotion-based reciprocity, where individuals choose to cooperate with partners with whom they associate positive emotions, which reduces the effective group size. Third, it is also possible that individuals switch between strategies dependent on their network size, i.e. using estimated scores when networks are large (generalised reciprocity) and more exact measurements when networks are small (direct reciprocity).

Most likely individuals memorise different partners using an Object File System (Kahneman, Treisman, & Gibbs, 1992). This cognitive system enables individuals to represent the exact numerical value of up to four entities or encounters. Individuals can represent more than just four entities, here however, the numerical value is approximated, rather than exact, i.e. Analogue Magnitude System (Kahneman et al., 1992). In reciprocal interactions, it is needed to encode several interactions or partners. The exact values of received and given help are not needed in emotion-based and attitudinal reciprocity, which can enable reciprocity with more than four partners. Hard-wired reciprocity, in contrast, most likely applies only to dyadic encounters. Given that calculated reciprocity requires the most information about recent encounters compared to all other mechanisms of reciprocity, it is most likely confined to fewer partners.

The memory capacity problem increases if individuals engage in indirect reciprocity. For instance, an individual that lives with ten partners needs to remember the behaviour of ten interactions in order to show direct reciprocity. However, to show indirect reciprocity, the individual needs to remember 45 interactions. Thereby, it might be important to encode not only whether the observed individual cooperated or defected, i.e. image strategy (Nowak & Sigmund, 1998), but also whether the interaction partner had cooperated or defected in a previous encounter (Sugden, 1986), resulting in at least 90 interactions. This shows that third-party evaluations can be challenging in terms of memory load and has been suggested to be a driver of brain size in primates (Dunbar, 1992; but see Healy & Rowe, 2007). Still, there is evidence that cleaner fish (*Labroides dimidiatus*) behave more cooperatively in the presence of bystanders (Pinto, Oates, & Grutter, 2011) and bystanders are more likely to approach cleaner fish with a good image (Bshary & Grutter, 2006). This suggests that large brains are not necessary for indirect reciprocity, which can be achieved by different mechanisms. However, indirect reciprocity is unlikely to be explained by calculated reciprocity, if individuals interact regularly with more than two partners and their decisions are based on interactions that are not distinct, emotional or require the memory of many details.

### Processing

In addition to how information is encoded and retrieved, reciprocity is dependent on how such information is processed in order to return favours. There are three systems possible. First, individuals may update old information with new information and thereby base their decisions to help on the most recent encounter. Second, individuals may accumulate received help into an overall score, which is the basis for their decision to help. Third, individuals may calculate and integrate how much a partner has helped them over a given period by adding and subtracting received and given help, in order to help reciprocally. The three systems may be either partner specific, i.e. direct and indirect reciprocity, or partner unspecific, i.e. generalised reciprocity.

Updating or replacing information is an integral part of attitudinal reciprocity and probably the simplest type of processing. Updating information is part of almost all animals’ life whenever new information takes precedence, for example when there is a better mating partner, a new group member, a different predator or a new food patch that has been discovered. Furthermore, many animals need to update information about specific partners as they may change over time by altering their acquired knowledge, hierarchical rank, network size, mating status, partner quality, physical strength, or their cooperation level. Hence, updating information, which is tightly linked to attitudinal reciprocity, lies most likely within the cognitive capabilities of many species.

Probably more demanding is accumulating information into one score. Here, information about a partner needs to be stored and integrated with existing information to produce an estimated score rather than calculating received and given help over multiple encounters. This could be achieved by associating a ‘cooperative’ tag with a partner. The more often the partners helped an individual, the stronger the association becomes without performing any operations on the amount of help, i.e. association model (reviewed in Gallistel & Gibbon, 2001). An accumulation could be also achieved by memorising single events that are accumulated by adding one cooperative event to another, i.e. computational model (reviewed in Gallistel & Gibbon, 2001). Here, individuals may have to potentially add large numbers of received help into a score, which can be demanding. Yet, some species have been shown to add even large numbers into an approximate score (Beran & Beran, 2004), which are most likely represented as approximate magnitudes (Kahneman et al., 1992). For instance, many mammals form social bonds, which are accumulated scores of positive interactions with specific partners (Massen et al., 2010; Seyfarth & Cheney, 2012). This shows that many mammals use partner’s specific scores in their interactions. Therefore, it seems reasonable to assume that at least those species may be capable of emotion-based reciprocity.

Finally, only calculated reciprocity requires computations, which is, however, within the scope of non-human animals. Many animals have been shown to perform at least simple calculations in terms of adding and subtracting (Beran, 2004; Cox & Tamara Montrose, 2016; Olmstead & Kuhlmeier, 2015b; Pepperberg, 2017; Potrich, Sovrano, & Vallortigara, 2015). These examples involve only small quantities of up to four items or encounters, and respective calculations can be explained by an Object File System because it requires exact calculations (Kahneman et al., 1992). It is possible that individuals remember specific episodes and integrate this information, for example 'my partner was cooperative in four episodes'. This integrated information can then be carried forward in a computationally accessible form (reviewed in Gallistel, 2008). This psychological process, which might be similar to path integration and cognitive maps, is likely to require less memory and computational demands as the exact details of past episodes may be forgotten (Lotem & Halpern, 2012). Processing information in order to reciprocate help, including exact calculations and summary integrations, can be influenced by the quantity and quality of encounters, which we discuss below.

### Processing and quantity

Decisions to help are dependent on how many encounters with a partner are used to reciprocate. A meta-analysis in non-human primates showed that only a small proportion of received help is paid back immediately (Schino & Aureli, 2016, see also Jaeggi, de Groot, Stevens, & van Schaik, 2013) and studies investigating immediate turn-taking have not yielded evidence for reciprocity (Melis, Grocke, Kalbitz, & Tomasello, 2016; Yamamoto & Tanaka, 2009). This indicates that help is usually reciprocated over long time intervals at least in non-human primates. During such intervals, it is likely that individuals meet the same or different partners repeatedly. Therefore, individuals potentially need to remember several encounters with single partners.

It is unlikely that non-human animals remember the exact details of more than four encounters (Kahneman et al., 1992). Studies on various species showed that individuals can only represent up to four items or encounters, which is operated by the Object File System (reviewed in Beran, Parrish, & Evans, 2015). If more than four encounters need to be memorised, the level of accuracy decreases and details are approximate rather than exact, which is operated by the Analogue Magnitude System. This is why many species can easily distinguish between two and three units, but not between five and seven (reviewed in Olmstead & Kuhlmeier, 2015b).

Only calculated reciprocity requires the exact details of multiple encounters. Consequently, calculated reciprocity might be constrained to four encounters. In contrast to calculated reciprocity, all other forms of reciprocity either update previous encounters with the most recent encounter, i.e. hard- wired and attitudinal, or accumulate positive emotions in an estimated score, i.e. emotion-based reciprocity. Thereby, exact details of received help during multiple encounters become less important or play no role at all. It is yet unclear how many details of encounters are needed to deploy the three behavioural strategies. The only strategies that has been investigated is direct reciprocity, which has been shown to be a stable strategy when one or two encounters with a partner are considered (Axelrod & Hamilton, 1981; Boyd & Lorberbaum, 1987).

### Processing and quality

Reciprocal decisions involve information not only about the quantity of help, i.e. providing approximately the same amount of received help (see above), but also about the quality of help, i.e. providing approximately the same value of received help. However, ensuring a balance of received and given help can be challenging, especially if exchanges involve different commodities, such as food for sex. Here, two possibilities are likely. Either animals consider the value of received help or not. In hard-wired reciprocity, commodities are usually fixed and there is no variation in commodities differing in quality. Hence the value is unlikely to influence reciprocity. All other mechanisms are likely to involve the assessment of quality and currency conversions to balance received and given help.

There is evidence that animals can assess and compare items of different values. For instance, brown capuchin monkeys are more likely to trade tokens of lower value with tokens that result in higher value food (Westergaard, Liv, Rocca, Cleveland, & Suomi, 2004). In addition, they protest if they exchange a token with an experimenter that results in a low-value food item, while the same token results in a high-value food item for a conspecific (Brosnan & de Waal, 2003). Furthermore, values may change. Pigeons, for instance, prefer tokens that result in food under food-restricted conditions but change their preference to tokens that result in water under water-restricted conditions. In addition, if given the choice between specialised and generalised tokens, which can be exchanged for multiple purposes, they opt for the latter more flexible option (Defulio, Yankelevitz, Bullock, & Hackenberg, 2014).

In calculated reciprocity, the value of help might be encoded as multiplications, for example food is twice as valuable as allogrooming. Adding value to such calculations increases the challenges associated with this mechanism, especially when individuals meet several times and information of multiple encounters might be confused. In emotion-based and attitudinal reciprocity, the value of help might be encoded in the strength of association, i.e. the more valuable the received help, the stronger the resulting positive association with this partner and the more or the better help will be provided in return. Although many animals show reciprocal exchanges between different services (e.g. Hemelrijk, 1994; Schweinfurth & Taborsky, 2018b; reviewed in Stevens & Gilby, 2004) and respond to help differing in value (Dolivo & Taborsky, 2015), no study of which we are aware has investigated the psychological processes of how animals use the value of help in their decisions to reciprocate.

## Predicting the occurrence of reciprocity

So far, the literature has focussed mainly on the psychological processes of calculated reciprocity with the result that reciprocity *per se* has been considered to be too cognitively demanding for non-human animals (see above). Here, we showed that calculated reciprocity requires a very specific set of cognitive capabilities that indeed only a few species may show. However, there are three other cognitive mechanisms that require less sophisticated skills. We propose that a more inclusive approach might be more fruitful that encompasses all forms of reciprocity and their specific psychological processes. Based on the mechanisms that we listed above, next we make predictions about the circumstances that would favour each reciprocal behavioural strategy and cognitive mechanism.

Calculated reciprocity is the most complex mechanism of reciprocity that also adult humans only rarely use (e.g. Carter, 2014). To date, there has been no convincing evidence of calculated reciprocity in non-human animals besides one orang-utan dyad (Amici et al., 2014; Dufour, Pelé, Neumann, Thierry, & Call, 2009; Pelé et al., 2009). Still, calculated reciprocity can occur, if individuals are able to remember few distinct encounters with few partners, which potentially induce an emotional response. Furthermore, individuals must be able to make computations like adding, subtracting and potentially multiplying units. Because individuals would have to remember many different interactions when applying indirect reciprocity, calculated reciprocity is most likely confined to direct reciprocity. Given that many details of encounters need to be stored, it is most likely that reciprocity is further confined to short time delays between received and given help. It might thus be possible that some species show calculated reciprocity only with unfamiliar partners where calculations are needed because no other additional information about the likelihood to reciprocate is available and calculations are possible because only few encounters have happened. However, the more often an individual meets a partner, the more likely they switch to another mechanism because it becomes difficult to remember several encounters in potentially similar situations. Therefore, an episodic memory might be replaced by a scene memory or the information might be accumulated into a social bonding score. We, therefore, predict that calculated reciprocity may be shown by animals that form social bonds with group members but that are also highly tolerant towards unknown individuals to direct affiliative behaviours to them according to direct reciprocity.

Emotion-based reciprocity requires less cognitive processing compared to computations, and reduced memory demands compared to episodic memory. Emotion-based reciprocity can underlie reciprocity in species that form social bonds, can experience emotions and are able to accumulate information into an estimated approximate score. This mechanism most likely results in direct reciprocity because decisions to help are based on emotions associated to directly experienced partners with which they have a positive social bond.

Attitudinal reciprocity further lowers the cognitive demands. Attitudinal reciprocity can underlie reciprocity, if individuals are able to remember single encounters that are partner specific, i.e. in/direct reciprocity, or unspecific, i.e. generalised reciprocity. In addition, individuals need to update old information with new information. Thereby an estimation, i.e. Analogue Magnitude System, rather than exact information, i.e. Object File System, is most likely sufficient for reciprocity. The memory of recent encounters with several partners may be enhanced but is not constrained to encounters that involve distinct situations, emotional reactions or short time delays. If generalised reciprocity is applied, the memory demands can be further lowered because only a single encounter needs to be remembered. We predict to find this mechanism in species that form groups in which they meet their group members repeatedly.

Hard-wired reciprocity requires no cognition or emotions and may explain reciprocity in gregarious individuals that are unlikely to meet often. Hard-wired reciprocity can underlie reciprocity, if individuals engage in automatic and immediate exchanges with one partner. Whether or not the information of the partner’s identity is integrated in this form of reciprocity is currently unknown and consequently generalised and direct reciprocity strategies may be used.

## Conclusions and future directions

It has been stated frequently that reciprocity is too cognitively demanding for non-human animals (Hauser et al., 2009; Ramseyer et al., 2006; Russell & Wright, 2009; Sánchez-Amaro & Amici, 2015; Stevens et al., 2005; Stevens & Gilby, 2004; Stevens & Hauser, 2004, 2005). However, this perception is based on the idea that there is only one form of reciprocity that is achieved by one mechanism, i.e. calculated reciprocity (cf. Carter, 2014; Schino & Aureli, 2010a). However, here we show that reciprocity encompasses at least three different strategies that can be underlain by at least four different mechanisms, that vary dramatically in its psychological processes. This shows that reciprocity is a more diverse phenomenon than currently appreciated and may not be too cognitively demanding for animals other than humans. Hence, some forms of reciprocity lay in the range of many species. We propose that acknowledging this diversity of strategies and mechanisms, which result in individuals exchanging goods and services that are contingent on each other, will inform us about the evolutionary and psychological origins of reciprocity.

Until now most information on different forms of reciprocity have come from a few species. Therefore, a first step would be to investigate different strategies and mechanisms in various species that fulfil the above described social and cognitive requirements. For example, to the best of our knowledge, only one empirical study has provided test subjects with conflicting partner experiences and therefore tested for the potential integration of several encounters (Schweinfurth & Taborsky, submitted). In this study, Norway rats based their decision to help on the last encounter. Given that this is the only study of this type so far, it is currently unclear how other non-human animals encode multiple encounters, which would give insight into the mechanism they are using to reciprocate. The same applies to different strategies. Only few studies have tested whether test subjects help conspecifics according to direct or generalised reciprocity, which cannot be distinguished in dyadic situations (but see: Carter & Wilkinson, 2013c; Gfrerer & Taborsky, 2017; Leimgruber et al., 2014; Majolo, Schino, & Aureli, 2012; Rutte & Taborsky, 2008). Furthermore, by collecting data on various species, we might be able to conduct phylogenetic analyses to test how different forms of reciprocity evolve and whether some might be basal to others (cf. MacLean et al., 2012).

In addition, brown capuchin monkeys and humans apply different reciprocal strategies with different mechanisms enabling them. This shows that species are not constrained to a single mechanism or a single strategy. Still, we understand little about the development of reciprocity, i.e. when individuals use which mechanism to reciprocate help and when they switch from one to another. A possible experiment would be to test partners that are unfamiliar or familiar with each other in a reciprocal paradigm. This would inform us about whether repeated actions are responsible for changing the mechanism of reciprocity. Observing the onset of a relationship would provide additional information on switching points, the developmental sequence of mechanisms and their connection to strategies. Another possible experiment could be to present test subjects with few or many partners in a reciprocal paradigm that involves few or many interactions with these partners. This would shed light on a flexible use of reciprocal strategies, for instance, based on group size.

In sum, reciprocity is not cognitively demanding *per se*. There are different forms of reciprocity, including different strategies and mechanisms. The psychological processes vary between these forms and we showed that complex calculations are not the only mechanisms through which reciprocity can be achieved. It is time to acknowledge the diversity of reciprocal interactions and aim at understanding their evolutionary and psychological origins.
